# Metal–Organic Framework Materials for Hydrogen Storage Applications

**DOI:** 10.3390/molecules31152643

**Published:** 2026-07-29

**Authors:** Yitong Liu, Shuyuan Chen, Dan Li, Teng Zhang, Yuanbo Wang

**Affiliations:** 1 Key Laboratory of Cluster Science Ministry of Education, Beijing Key Laboratory of Photoelectronic/Electrophotonic Conversion Materials, Advanced Research Institute of Multidisciplinary Science, School of Chemistry and Chemical Engineering, Beijing Institute of Technology, Beijing 100081, China; 3220241956@bit.edu.cn (Y.L.);; 2 Advanced Technology Research Institute (Jinan), Beijing Institute of Technology, Jinan 250101, China

**Keywords:** metal–organic frameworks, hydrogen storage, porous materials, hydrogen storage mechanism

## Abstract

Hydrogen, as a clean and renewable energy carrier, offers a promising solution to the global energy challenge, yet its safe and efficient storage remains a critical bottleneck. Metal–organic frameworks (MOFs), with their ultrahigh surface area, tunable porosity, and excellent stability, have emerged as leading candidates for physical hydrogen storage. This review systematically surveys recent progress in MOF-based hydrogen storage, organized by metal center type and examines the distinct adsorption mechanisms that govern hydrogen uptake. The regulatory effects of critical parameters including metal ion selection, pore architecture, and ligand functionalization on hydrogen storage capacity are analyzed in detail. Beyond material-level discussion, this review discusses the potential of MOFs for cryo-compressed hydrogen storage conditions. Key challenges facing practical deployment, including synthesis scalability, structural stability under cryogenic high-pressure cycling, and the knowledge gap in multi-cycle temperature-swing stability, are critically assessed. The roles of computational simulations and machine learning in accelerating MOF discovery and high-throughput screening are also reviewed. Finally, an application-oriented outlook is presented, mapping MOF performance to three specific industrial scenarios with reference to relevant economic analyses, thereby bridging fundamental materials chemistry with practical engineering requirements.

## 1. Introduction

Hydrogen gas can be used as a clean energy source due to its relatively high energy density. The combustion calorific value of hydrogen gas is 142.35 kJ/g, which is approximately 2.7 times that of gasoline and 4 times that of coal. Hydrogen combustion only produces water, enabling the design of environmentally friendly processes that avoid the emission of pollutants such as carbon dioxide, nitrogen oxides, or sulfides [1]. Furthermore, hydrogen energy is renewable and can be produced via water splitting through the decomposition of water using natural resources such as wind and solar power [2,3]. Therefore, hydrogen shows excellent promise for applications in various sectors, including energy, transportation, industry, and construction [4]. However, despite the numerous advantages of hydrogen energy, the storage and transportation of hydrogen are still highly challenging, which has limited its commercialization [5,6].

**Figure 1:**
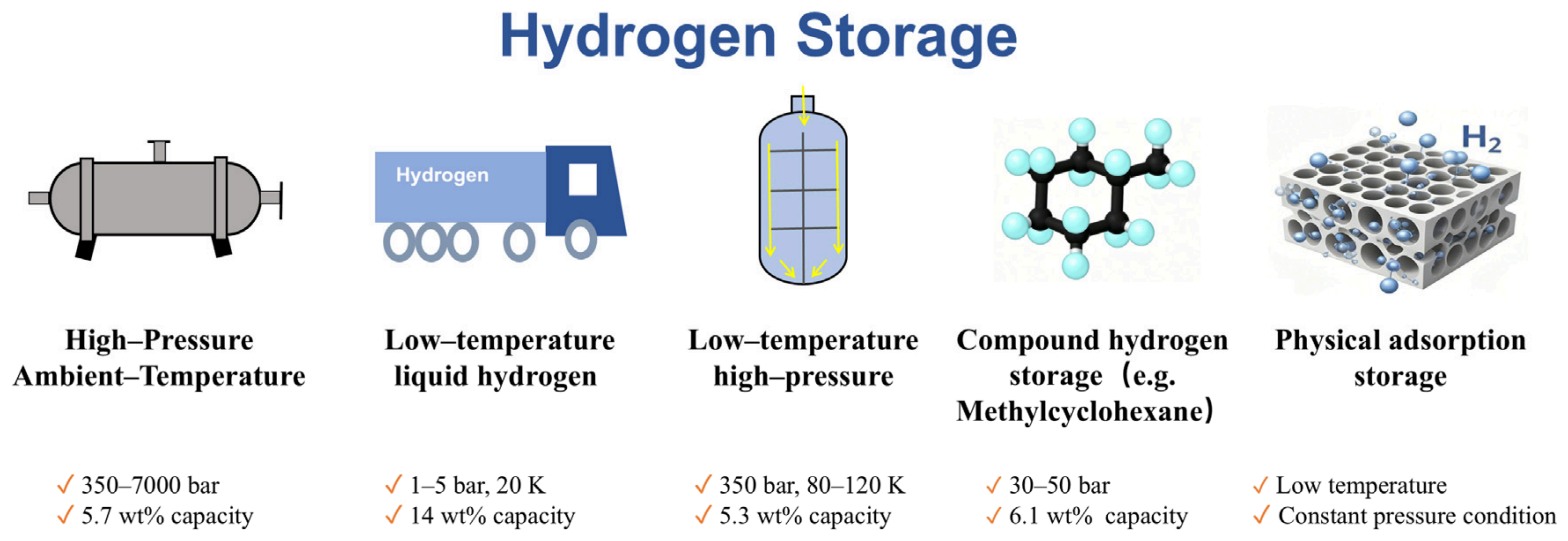
Overview of typical hydrogen storage technologies.

Since 2005, the U.S. Department of Energy (DOE) has released hydrogen storage targets every five years [7], with the ultimate long-term targets including a gravimetric capacity of 6.5 wt%, a volumetric density of 50 g H_2_/L, an operating temperature range of −40 to 85 °C, and a delivery pressure of 5–12 bar, corresponding to an optimal hydrogen binding energy of 0.2–0.7 eV [8]. Currently, the primary hydrogen storage and transportation methods include high-pressure gaseous storage at ambient temperature, low-temperature liquid hydrogen storage, low-temperature high-pressure (cryo-compressed) hydrogen storage, compound storage, and physical adsorption-based storage. High-pressure gaseous storage is a relatively mature and cost-effective technique. Nevertheless, this storage approach necessitates the use of containers capable of withstanding high pressure and suffers from low storage density, thereby constraining its suitability for large-scale or long-duration applications [9]. Cryogenic liquid hydrogen storage has a high volumetric hydrogen storage density and low storage pressure, but the energy consumption for hydrogen liquefaction is high, accounting for more than 30% of the hydrogen’s calorific value. The low storage temperature of liquid hydrogen imposes stringent insulation performance requirements on storage containers. Additionally, hydrogen liquefaction and storage equipment are complex, require a large initial investment, have high maintenance and support demands, and poor operational flexibility [10]. Low-temperature high-pressure hydrogen storage combines two densification techniques: increasing pressure and lowering temperature. The hydrogen storage density can reach that of liquid hydrogen. The storage temperature is higher (in the 80–120 K range) and does not require ortho-para hydrogen conversion [11]. Its intrinsic energy consumption is significantly lower than hydrogen liquefaction, eliminating the need for refrigeration and storage equipment at the 20 K temperature range. This results in lower safety requirements and better equipment investment and operational economics [12]. Low-temperature high-pressure hydrogen storage still requires relatively high pressure to achieve higher hydrogen storage density, posing severe challenges to the preparation system and hydrogen storage containers. Despite these challenges, cryo-compressed hydrogen storage technology has been recognized as a strategic priority in the hydrogen development roadmaps of major economies worldwide [13,14,15]. Compound hydrogen storage involves chemically reacting hydrogen gas to form stable hydrogen-containing compounds with materials in atomic or ionic form, achieving high-density and safe storage—for instance, MgH_2_, LiBH_4_, and NaAlH_4_ offer theoretical capacities of 7.6, 18.5, and 7.4 wt% [16], respectively—meeting or exceeding the DOE gravimetric target. However, these systems require elevated temperatures (typically 200–400 °C) for hydrogen release and incur significant system-level weight penalties, limiting their practical applicability. Physical adsorption storage methods employ porous or layered materials such as carbon, metals, or organic networks. These materials possess large specific surface areas and high porosities, enabling the effective physical adsorption of hydrogen molecules [10,17]. Physical adsorption hydrogen storage has the advantages of fast hydrogen adsorption and desorption rates, good cyclic reversibility, and mild operating conditions, but the weak physisorption enthalpy restricts room-temperature capacities to well below the DOE target. Developing materials that can reversibly store hydrogen under mild conditions while approaching the DOE targets therefore remains a major challenge [18,19]. Figure 1 provides a comparative schematic overview of these five typical hydrogen storage technologies, illustrating their respective operational mechanisms, temperature-pressure ranges, and key performance characteristics [2]. A variety of porous materials, such as metal–organic frameworks (MOFs), covalent organic frameworks (COFs), activated carbon (AC), and zeolites, have attracted a lot of interest due to their potential for effective hydrogen storage [20]. Table 1 presents a comparative analysis of representative porous adsorbents from various material classes, including MOFs, COFs, activated carbons, and zeolites, assessed under cryogenic conditions. MOFs, characterized by their exceptionally high specific surface areas and precisely tunable pore architectures, have emerged as the most promising materials for physical adsorption-based hydrogen storage applications [18,21,22].

**Figure 2:**
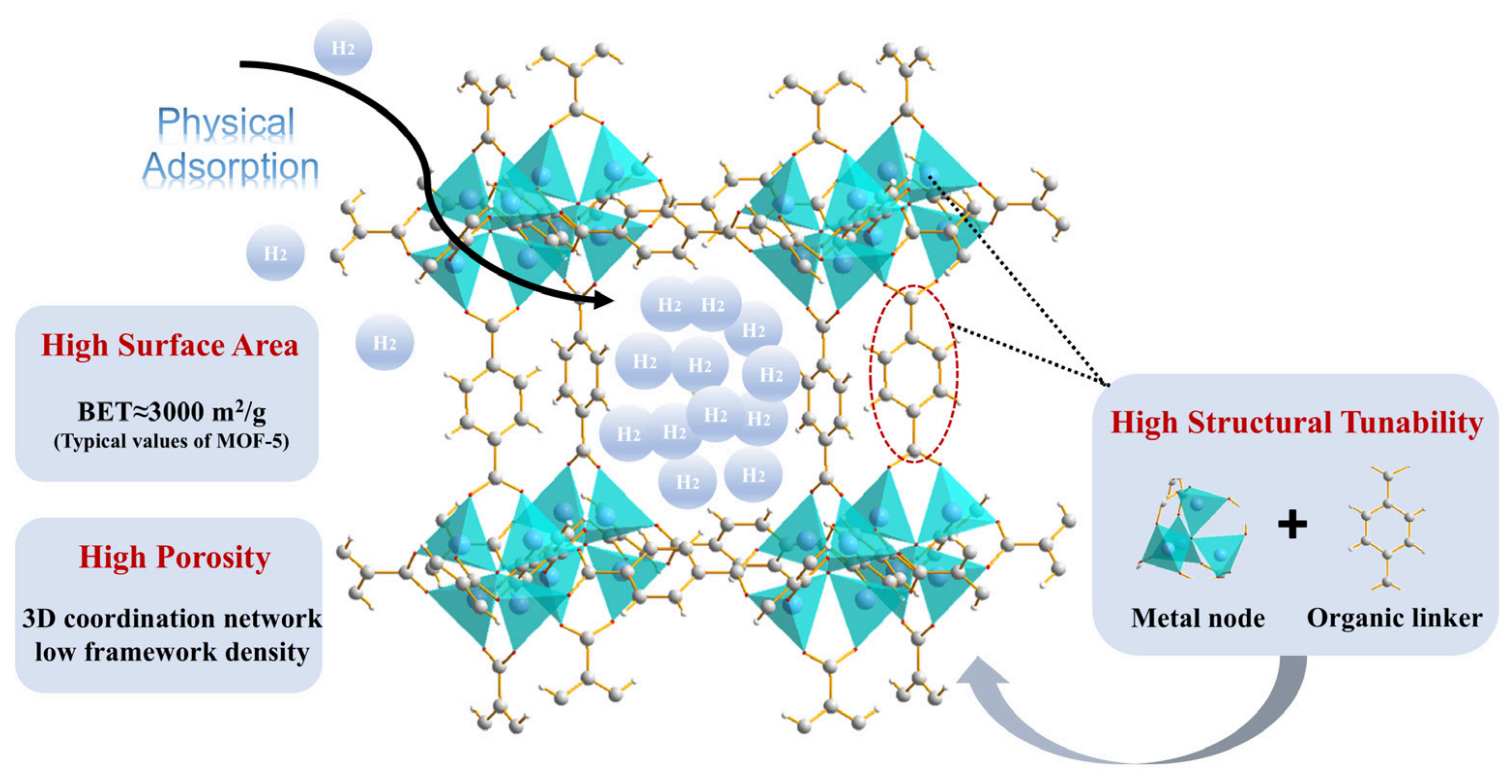
Schematic of MOFs (MOF-5 as representative): Structure and physical H_2_ storage advantages. Cyan polyhedra stand for Zn_4_O metal nodes, while gray spheres and yellow bonds constitute organic linkers. Pale blue spheres represent adsorbed H_2_ molecules within MOF pores.

MOFs represent an innovative category of porous solid materials synthesized through the self-assembly of metal ions or clusters coordinated with organic ligands. These materials exhibit crystalline architectures characterized by exceptionally high surface areas and significant porosity. They also have customizable pore sizes and abundant pore space for hydrogen adsorption (Figure 2). Because the crystal structures of MOFs are particularly well-suited for accommodating guest molecules and can effectively adsorb hydrogen through physical interactions, MOFs have become a focal point in hydrogen storage research and development [32,33,34,35,36,37,38]. The hydrogen storage capabilities of MOFs are fundamentally influenced by the interactions between hydrogen molecules and the material’s surface. In addition to the common physisorption driven by van der Waals forces, MOFs exhibit further adsorption mechanisms—primarily involving the hydrogen spillover effect and Kubas-type interactions—that can enhance hydrogen uptake under specific conditions. The hydrogen spillover effect describes the process whereby catalyst nanoparticles facilitate the chemisorption and dissociation of molecular hydrogen, enabling the subsequent diffusion of atomic hydrogen onto the support surface. In this context, Kang et al. decorated Pt nanoparticles onto amino-functionalized UiO-66 and achieved a hydrogen storage capacity of 0.71 wt% at 300 K and 30 bar—nearly an order of magnitude higher than the undoped material (0.08 wt%)—by enabling H_2_ dissociation on the Pt surface followed by migration of atomic hydrogen to the MOF support [39]. Hu et al. employed an ultrasound-assisted double-solvent method to confine Pd nanoparticles within the pores of Cu-based HKUST-1, yielding a room-temperature hydrogen uptake of 1.63 wt% at 180 bar through spillover-mediated adsorption [40]. The Kubas interaction represents a distinctive form of weak intermolecular bonding characterized by the elongation of the H–H bond without its cleavage. This bond elongation results from two cooperative mechanisms: the σ bonding orbital of the hydrogen molecule donates electron density to the vacant d orbital of the metal center (σ donation), while concurrently, the empty σ* antibonding orbital of H_2_ accepts π back-donation from an occupied d orbital of the metal. The incorporation of unsaturated metal sites has been shown to enhance the Kubas interaction, thereby increasing the adsorption enthalpy in MOFs. A representative example is the Mn-based MOF Mn-BTT (BTT^3−^ = 1,3,5-benzenetristetrazolate), which possesses a high density of unsaturated Mn^2+^ sites and achieves a hydrogen storage capacity of 0.95 wt% at ambient temperature and 90 bar via Kubas-type interactions [41]. Due to the weak interaction between hydrogen and MOFs, most studies have evaluated hydrogen storage performance at low temperatures. Recent advances have emphasized the unique synergy between porous adsorbents and cryo-compressed hydrogen storage conditions. Wang et al. demonstrated through thermodynamic analysis that a mixed-refrigerant Joule–Thomson cycle can efficiently cool compressed hydrogen to the 80–120 K range with significantly lower intrinsic energy consumption compared to hydrogen liquefaction, establishing a practical thermal foundation for coupling MOFs with cryo-compressed systems [42]. A comprehensive review by Wang et al. further highlighted that introducing adsorbent materials such as MOFs into cryo-compressed hydrogen storage vessels can substantially enhance the hydrogen storage density while reducing the required operating pressure, thereby alleviating the demands on vessel wall thickness and the cooling system [43]. This material-process coupling strategy is particularly well-suited to MOFs, whose high specific surface area and tunable pore architecture enable dense hydrogen packing through enhanced van der Waals interactions at cryogenic temperatures, making them the most promising adsorbent candidates for this operating regime.

This review provides a comprehensive survey of recent progress in hydrogen storage research, with particular emphasis on the distinct hydrogen storage mechanisms exhibited by various metal-based MOFs. The regulatory influence of critical parameters—including metal ions, pore architecture, and ligand functionalization—on hydrogen storage capabilities is systematically examined. Beyond laboratory-scale material characterization, this review discusses the prospects of MOFs for cryo-compressed hydrogen storage, a regime where the combination of enhanced van der Waals interactions and dense molecular packing under elevated pressure compensates for the inherently weak physisorption enthalpy of MOFs. The present work further identifies key knowledge gaps, notably the lack of standardized multi-cycle temperature-swing stability testing protocols essential for practical operation. The roles of computational simulations and machine learning in accelerating the discovery of MOFs with targeted pore environments are also discussed, bridging the gap between theoretical prediction and experimental realization. Finally, an application-oriented framework is presented, mapping MOF performance to three specific industrial scenarios with reference to relevant economic considerations for cryo-compressed hydrogen infrastructure, thereby connecting fundamental materials chemistry with practical engineering requirements.

## 2. Metal–Organic Frameworks

### 2.1. Research Progress in the Utilization of MOFs as Hydrogen Storage Materials

#### 2.1.1. Zn-Based MOFs

Zn-based MOFs prepared by combining a transition metal Zn precursor with organic ligands have been extensively studied for hydrogen storage applications. MOF-5, which is prepared using Zn^2+^ ions and phthalic acid ligands, is a typical representative example of Zn-based MOFs. In 2003, Yaghi et al. first reported that MOF-5 could be used for hydrogen storage, demonstrating that the material can adsorb approximately 4.5 wt% hydrogen at 78 K and 1 bar [23]. In addition, at mild temperature and 20 bar, 1.0 wt% hydrogen could still be adsorbed. This work triggered a wave of subsequent studies on the use of MOFs as hydrogen storage materials.

**Figure 3:**
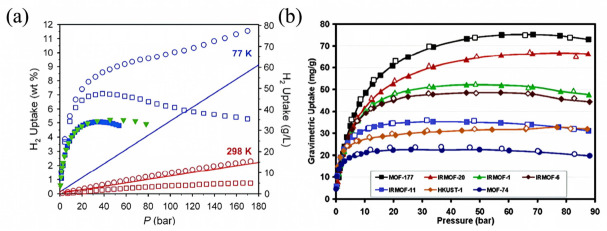
(**a**) Hydrogen adsorption isotherms measured at 77 K (blue) and 298 K (red), excess hydrogen storage volumes (squares), and total absorption volumes (circles) of optimized MOF-5. Solid squares: excess H_2_ uptake for air-exposed MOF-5; green triangles: excess H_2_ uptake for air-free high-quality MOF-5; open circles: total H_2_ uptake [44]. Reproduced with permission from Wong-Foy, A. G., et al., *J. Am. Chem. Soc.*; published by American Chemical Society, 2007. (**b**) High-pressure H_2_ isotherms for activated materials at 77 K in gravimetric units (mg/g) representing surface excess adsorption (excess adsorption refers to the amount of gas adsorbed on the surface, excluding the gas occupying the pore volume at the same temperature and pressure) Solid symbols = adsorption; open symbols = desorption [24]. Reproduced with permission from Kaye, S. S., et al., *J. Am. Chem. Soc.*; published by American Chemical Society, 2006.

Numerous investigations have been dedicated to enhancing the hydrogen storage capabilities of MOF-5. Kaye et al. [44] refined the synthesis protocol of MOF-5 by limiting its exposure to moisture and atmospheric air, as well as reducing crystal defects, thereby producing a material exhibiting an increased specific surface area. They evaluated the impact of air exposure on the material’s performance by using hydrogen adsorption isotherms. The optimized MOF-5 demonstrated an excess hydrogen storage capacity of 7.1 wt% at 77 K and 40 bar (Figure 3a). These findings substantiate that the actual hydrogen storage capacity of MOF-5 is much higher than early reports indicated, with the key being strict synthesis and activation under anhydrous and oxygen-free conditions. Lee et al. [45] incorporated platinum (Pt) and activated carbon (AC) into MOF-5 frameworks to synthesize MOF-5-based composites. They have determined that the hydrogen storage capacity of platinum–activated carbon at 298 K and 100 bar is approximately double that of unmodified activated carbon. Furthermore, under identical conditions, the hydrogen storage capacity of the platinum–activated carbon-MOF-5 composite attained 2.3 wt%, which is roughly three times greater than that of the platinum–activated carbon alone. Yang et al. [46] achieved a significant improvement in the low-temperature, low-pressure hydrogen storage performance of MOF-5 through structural regulation. Two typical modified derivatives were designed in this work: I-MOF (interpenetrated pure MOF-5 with double interwoven frameworks) and N-MOF (interpenetrated MOF-5 composite with acid-treated multiwalled carbon nanotubes, MWCNTs). Among them, the interpenetrated structure I-MOF reached a gravimetric hydrogen storage capacity of 1.7 wt% at 77 K and 1 bar, while the MWCNTs composite interpenetrated structure N-MOF achieved 2.0 wt% under the same conditions, representing an increase of approximately 67% compared with the original low-crystallinity P-MOF (pristine MOF-5, 1.2 wt%). The integration of interpenetration with carbon composites can inform the design of high-performance MOFs. Aljundi et al. [47] studied the incorporation of divalent metal ions into MOF-5 by using a post-synthesis exchange (PSE) method to prepare the mixed metal–organic framework M-MOF-5 (where M = Ni^2+^, Co^2+^, or Fe^2+^). The hydrogen adsorption capacities of Ni-MOF-5, Co-MOF-5, and Fe-MOF-5 at 77 K and 1 bar were 1.53 wt%, 1.53 wt%, and 0.99 wt%, respectively, while that of the original MOF-5 was 1.46 wt%. The incorporation of transition metal ions can strengthen the interaction with hydrogen molecules through modulation of the metal–organic framework structure.

In addition to MOF-5, researchers have also developed other Zn-based MOFs by combining the Zn_4_O(CO_2_)_6_ cluster with different organic ligands. For example, MOF-177 is synthesized using 1,3,5-tris(4-carboxyphenyl)benzene (H_3_BTB) as the ligand. Yaghi et al. reported that MOF-177 is capable of attaining a hydrogen storage capacity of 7.5 wt% at a temperature of 77 K and pressures ranging from 70 to 80 bar [24,48] (Figure 3b). At 298 K and 100 bar, this MOF had a hydrogen adsorption capacity of 0.62 wt%. Meanwhile, researchers improved MOF-177 by adding a catalyst that helps break hydrogen molecules apart and creating carbon links to promote hydrogen spillover. These tweaks boosted its ability to store hydrogen by roughly two and a half times, reaching about 1.5 wt%. These results indicate that MOF-177 is one of the most promising porous materials for high-capacity hydrogen storage at ambient temperatures. Furukawa et al. [25] further expanded this approach by synthesizing four additional Zn-based MOFs: MOF-180, MOF-200, MOF-205, and MOF-210. Among these, MOF-210, which was prepared using 1,3,5-tris(4-carboxyphenylacetylene)benzene and biphenyl dicarboxylic acid as ligands, possessed the highest specific surface area. At 77 K and 60 bar, MOF-210 demonstrated a total hydrogen capacity of 17.6 wt%. Han et al. [49] were the first to explicitly elucidate the fundamental discrepancy between total hydrogen storage capacity and the practically deliverable hydrogen amount. While the total hydrogen storage capacity of alkali metal-doped zeolitic imidazolate frameworks (ZIFs) increases in accordance with the ion binding energy sequence (Li^+^ > Na^+^ > K^+^), the practical deliverable hydrogen storage capacity exhibits an inverse trend (Na^+^ > K^+^ > Li^+^). This occurs because Li^+^ binds H_2_ too strongly to release fully at discharge pressure, while K^+^ binds too weakly to achieve sufficient uptake at charge pressure. Na^+^, with intermediate binding energy, provides the optimal balance. Related work by Mulfort and Hupp further showed that alkali metal doping can also enhance H_2_ uptake through structural effects, such as framework displacement increasing microporous surface area [50]. Panchariya et al. [51] demonstrated that employing a core–shell zeolitic imidazolate framework (ZIF) architecture constitutes an effective approach to improve the hydrogen storage capacity of ZIF materials under low-temperature and ambient-pressure conditions. Specifically, the ZIF-8@ZIF-67 composite exhibited a hydrogen storage capacity of 2.03 wt% at 77 K and 1 bar, representing a 41% enhancement relative to the original ZIF-8 material. Table 2 shows some examples of the hydrogen storage performance of various Zn-based MOFs under different temperatures and pressures.

#### 2.1.2. Cu-Based MOFs

**Figure 4:**
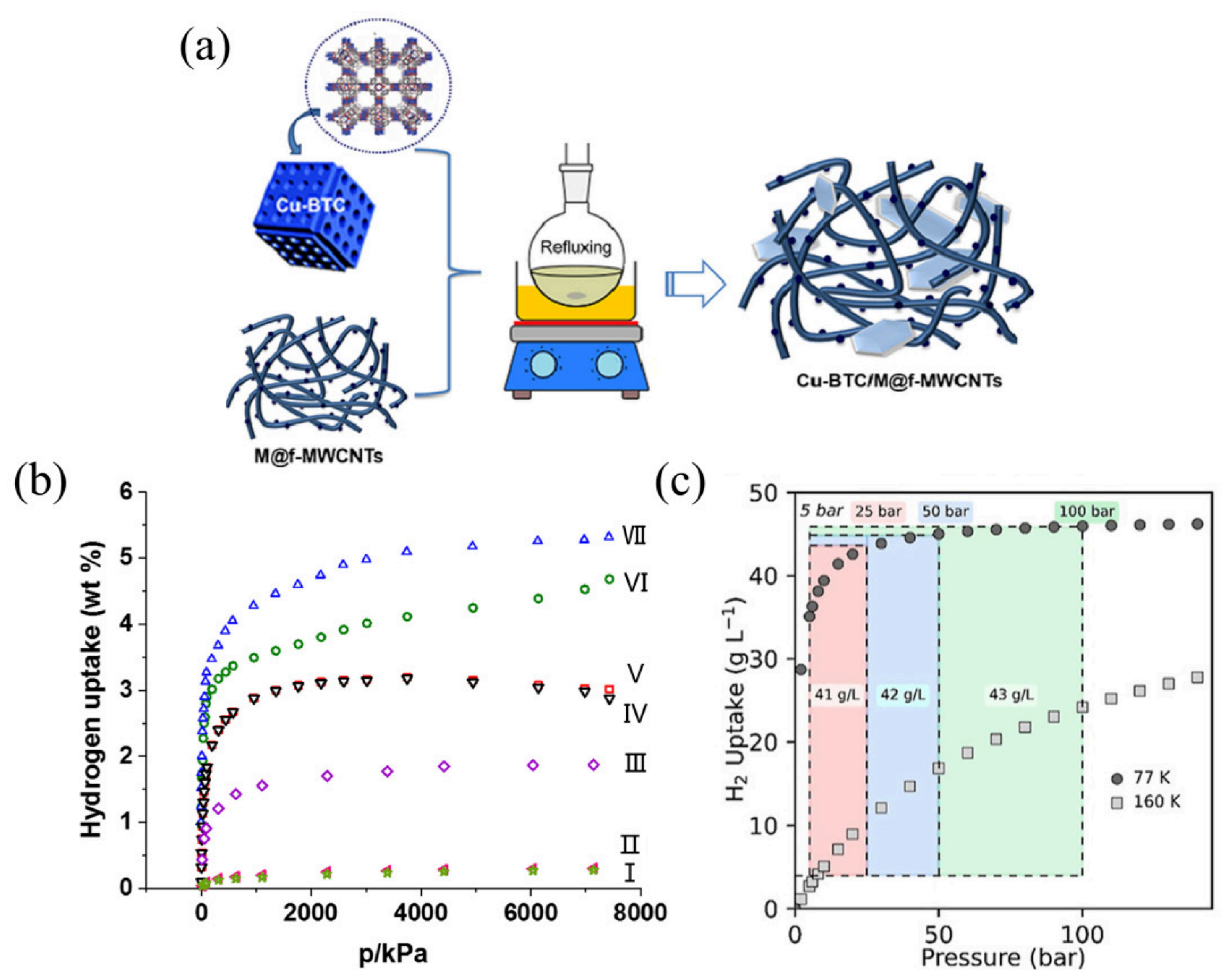
(**a**) Schematic illustration of the preparation of Cu-BTC/Ni@f-MWCNTs and Cu-BTC/Pd@f-MWCNTs [54]. Reproduced with permission from Konni, M., et al., *Surf. Interf.*; published by Elsevier, 2020. (**b**) Hydrogen uptake capacities of I p-MWCNTs, II f-MWCNTs, III Ni@f-MWCNTs, IV Pd@f-MWCNTs, V Cu-BTC, VI Cu-BTC/Ni@f-MWCNTs, and VII Cu-BTC/Pd@f-MWCNTs at 77 K [54]. Note: 100 kPa = 1 bar. Reproduced with permission from Konni, M., et al., *Surf. Interf.*; published by Elsevier, 2020. (**c**) Cryogenic H_2_ gas delivery for the temperature–pressure swing (100 bar/77 K → 5 bar/160 K) storage system [56]. Reproduced with permission from Madden, D. G., et al., *J. Am. Chem. Soc.*; published by American Chemical Society, 2022.

In Cu-based MOFs, copper ions serve as the central metal nodes. A prototypical example of such materials is HKUST-1 (Cu-BTC, where BTC denotes benzene-1,3,5-tricarboxylate), which consists of paddlewheel copper dimer secondary building units coordinated with trimesic acid ligands. The HKUST-1 sample synthesized by Panella et al. [52] exhibits a specific surface area of 1154 m^2^/g and demonstrates a hydrogen storage capacity of 3.6 wt% at 77 K and 80 bar. Furthermore, Li et al. [53] developed a Pd@HKUST-1 composite by encapsulating palladium nanocrystals within the HKUST-1 framework. The synergistic interaction between Pd and Cu sites in this composite resulted in a 74% enhancement in hydrogen storage capacity. This improvement is attributed to a combined mechanism involving Pd-catalyzed hydrogen spillover and Kubas-type interactions at the copper sites. Konni et al. [54] introduced an innovative composite nanomaterial integrating Cu-BTC with metal-functionalized multi-walled carbon nanotubes (Ni@f-MWCNTs or Pd@f-MWCNTs). Under experimental conditions of 77 K and 70 bar, the Cu-BTC/Ni@f-MWCNTs and Cu-BTC/Pd@f-MWCNTs composites demonstrated hydrogen storage capacities of 4.68 wt% and 5.31 wt%, respectively. At 298 K and 70 bar, their adsorption capacities were measured at 1.29 wt% and 1.67 wt%, respectively (Figure 4a,b). The superior performance of this composite material is attributed to the high electrical conductivity of the MWCNTs, which promotes efficient charge transfer, coupled with the presence of metal nanoparticles that serve as active adsorption sites.

Varghese et al. [55] developed an in-situ grown hybrid material consisting of Cu-BTC and an ultra-low 1 wt% content of graphene oxide (GO). In this Cu-BTC/GO structure, GO was employed as a structural guiding unit, forming new micropores on the Cu-BTC surface and enhancing the total pore volume and specific surface area. At 298 K, Cu-BTC/GO achieved hydrogen storage capacities of 0.46 wt% at 1 bar and 0.49 wt% at 3 bar, while those of pure Cu-BTC were 0.29 wt% and 0.35 wt%, respectively. Notably, the hydrogen adsorption capacity of Cu-BTC/GO rapidly increased at pressures below 1 bar. After adsorption–desorption experiments at 298 K and 1 bar involving six PSA cycles, both the Cu-BTC/GO and Cu-BTC adsorbents almost completely retained their initial hydrogen storage capacities.

Madden et al. [56] used GCMC simulations to investigate the hydrogen adsorption performance of four Cu-MOFs (MOF-199, MOF-399, PCN-20, and PCN-6′) with identical secondary building units (SBUs) under varying temperatures and pressures. PCN-6′ exhibited superior hydrogen adsorption capacities of 8.3 wt% at 77 K and 1.03 wt% at 298 K, and they concluded that MOF materials with smaller pore diameters and larger specific surface areas generally demonstrate enhanced hydrogen adsorption performance (Figure 4c).

Various other Cu-based MOFs have also been reported. For instance, Krawiec et al. [57] introduced a pressure-free synthesis method to prepare the porous material Cu_3_(BTC)_2_. This material exhibited an exceptionally high micropore volume of up to 0.62 cm^3^/g and achieved a hydrogen storage capacity of 2.18 wt% under low-temperature conditions. Yan et al. [58] used interphenyl dicarboxylate linkers and Cu(II) units to prepare a series of MOFs featuring a tetracarboxylate framework and open Cu(II) sites that strongly interacted with hydrogen gas. Among these MOFs, NOTT-103 demonstrated the highest hydrogen storage capacity of 7.78 wt% at 77 K and 60 bar. Sengupta et al. [59] proposed a novel synthetic approach for preparing NU-2100, an air-stable Cu(I)-based MOF material. NU-2100 was created using a mixture of copper and zinc precursors, with zinc acting as a catalyst to convert the intermediate MOF material into NU-2100. Notably, the material exhibited a gravimetric deliverable capacity of 4.8 wt% under a temperature and pressure swing from 233 K/100 bar to 296 K/5 bar. Chai et al. [60] constructed Cu&Li-based MOFs through structural optimization and Li^+^ modification. GCMC simulations demonstrated that the incorporation of Li^+^ ions induces substantial modifications in both the hydrogen adsorption capacity and adsorption characteristics across varying pressures and temperatures. Specifically, at 218 K and 0.1 bar, the presence of Li^+^ resulted in increases in adsorption capacity of 56.52% and 47.59%, respectively, with significant enhancements also detected at ambient temperature. These findings showed that adding Li^+^ caused noticeable changes in how much hydrogen could be adsorbed and how the adsorption behaved at different pressures and temperatures. Table 3 shows some examples of the hydrogen storage performance of various Cu-based MOFs under different temperatures and pressures.

#### 2.1.3. Zr-Based MOFs

Cavka et al. [61] pioneered the synthesis of Zr-based MOFs in their report introducing the UiO-66 framework. Using ZrCl_4_ as the metal precursor, benzoic acid (BDC) as the organic ligand, and dimethylformamide (DMF) as the solvent, MOFs with a high surface area and exceptional stability were successfully synthesized. Building on this work, Zhao et al. [62] synthesized UiO-66 at a low temperature of 323 K. They reported that pure-phase UiO-66 was only able to be synthesized in DMF, and this UiO-66 sample had a specific surface area of 1358 m^2^/g. Moreover, when comparing the hydrogen storage capacity of this UiO-66 material, it performed notably better under high-pressure and medium-pressure conditions, especially at 77 K and 18 bar, where it achieved an adsorption capacity of 3.35 wt%. Bambalaza et al. [63] enhanced the volumetric hydrogen storage capacity of UiO-66 by compressing its powder form. The densified UiO-66 retained 98% of its original surface area and porosity after compression at 7000 bar, achieving a total hydrogen storage capacity of 5.1 wt% at 100 bar and 77 K. Chen et al. [64] examined the effects of different reaction conditions on the structure and hydrogen storage performance of UiO-66(H_2_ADC). UiO-66(H_2_ADC)-SS was synthesized via a simple solution method, while UiO-66(H_2_ADC)-S was prepared using a solvothermal reaction method. Notably, the solvothermal synthesis method resulted in a larger specific surface area and a smaller average pore diameter of 2.73 nm. At 298 K and 50 bar, UiO-66(H_2_ADC)-SS and UiO-66(H_2_ADC)-S exhibited hydrogen adsorption capacities of 1.09 wt% and 2.99 wt%, respectively.

**Figure 5:**
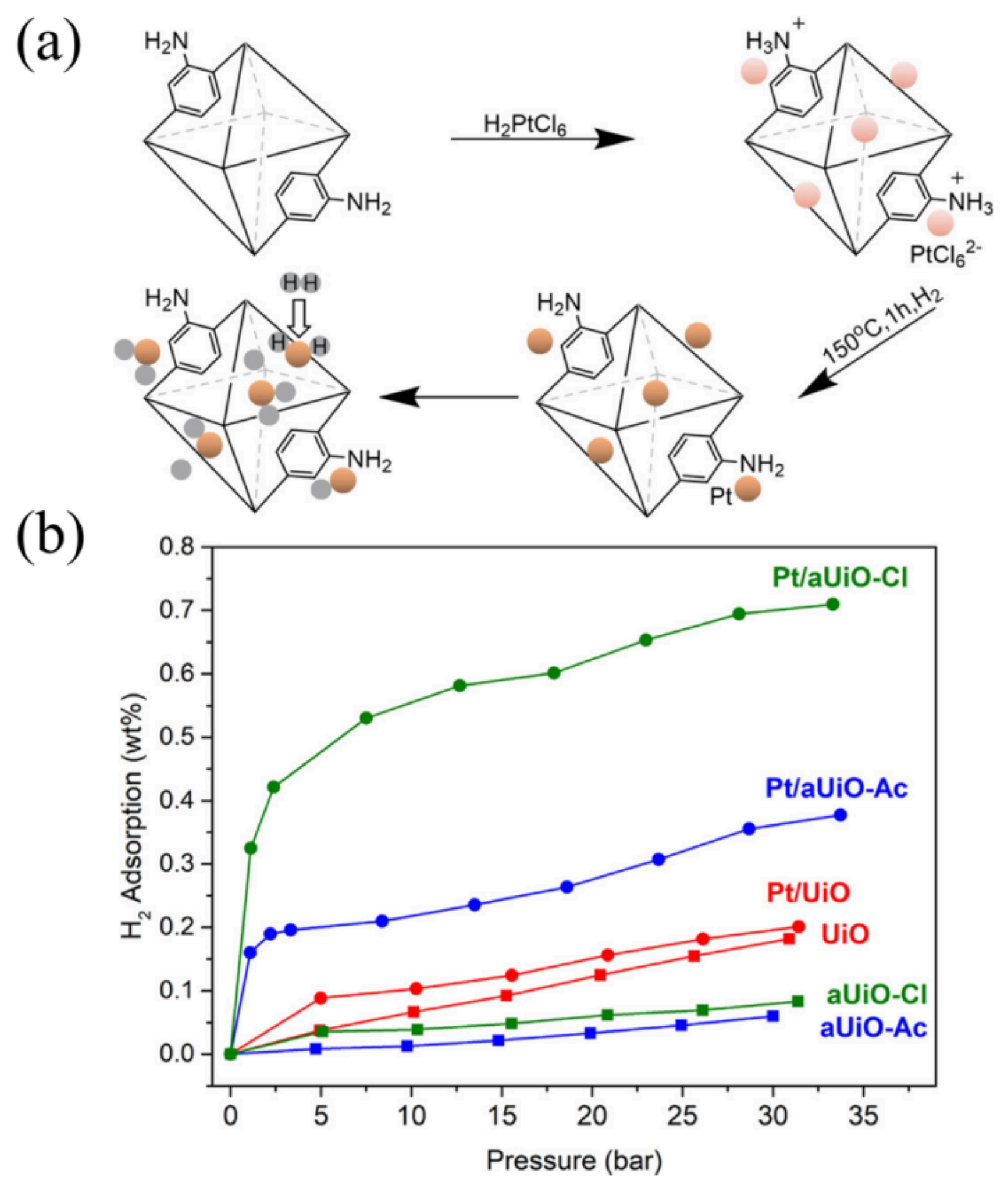
(**a**) Schematic representation of Pt/aUiO synthesis employing amino decoration to anchor platinum. (**b**) Hydrogen adsorption isotherms of pure MOFs (UiO, aUiO-Ac, and aUiO-Cl) [39]. Reproduced with permission from Kang, P.-C., et al., *ACS Appl. Nano Mater.*; published by American Chemical Society, 2021.

Kang et al. [39] synthesized UiO-66 and amino-functionalized UiO-66 (UiO-66-NH_2_) MOFs, then doped platinum (Pt) nanoparticles (NPs) onto the sample surfaces to obtain Pt/UiO, Pt/aUiO-Ac, and Pt/aUiO-Cl. The Pt/aUiO-Ac and Pt/aUiO-Cl materials achieved significantly enhanced hydrogen adsorption capacities of 0.38 wt% and 0.71 wt% at 34 bar and 33 bar, respectively (Figure 5). The Pt NPs provided more H_2_ adsorption sites and catalyzed the dissociation of H_2_.

Vaddanam et al. [65] synthesized three UiO-66-MOFs. UiO-66-AO@Si had a high specific surface area and pore volume. At 2.1 bar and 77 K, the hydrogen adsorption capacity of this MOF material (0.35 wt%) was higher than that of UiO-66-SO_3_H@Si (0.12 wt%) and UiO-66-PDCA (0.25 wt%). Liu et al. [66] introduced low-cost transition metal (Cu, Ni, and Cu-Ni alloy) nanoparticles into UiO-66 to improve its mild-temperature hydrogen storage performance. At 298 K and 60 bar, the maximum hydrogen storage capacity of the original UiO-66 was 0.20 wt%. In contrast, under the same conditions, the metal-doped Cu@UiO-66, Ni@UiO-66, and CuNi@UiO-66 MOFs had hydrogen storage capacities of 0.26 wt%, 0.45 wt%, and 0.74 wt%, respectively.

**Figure 6:**
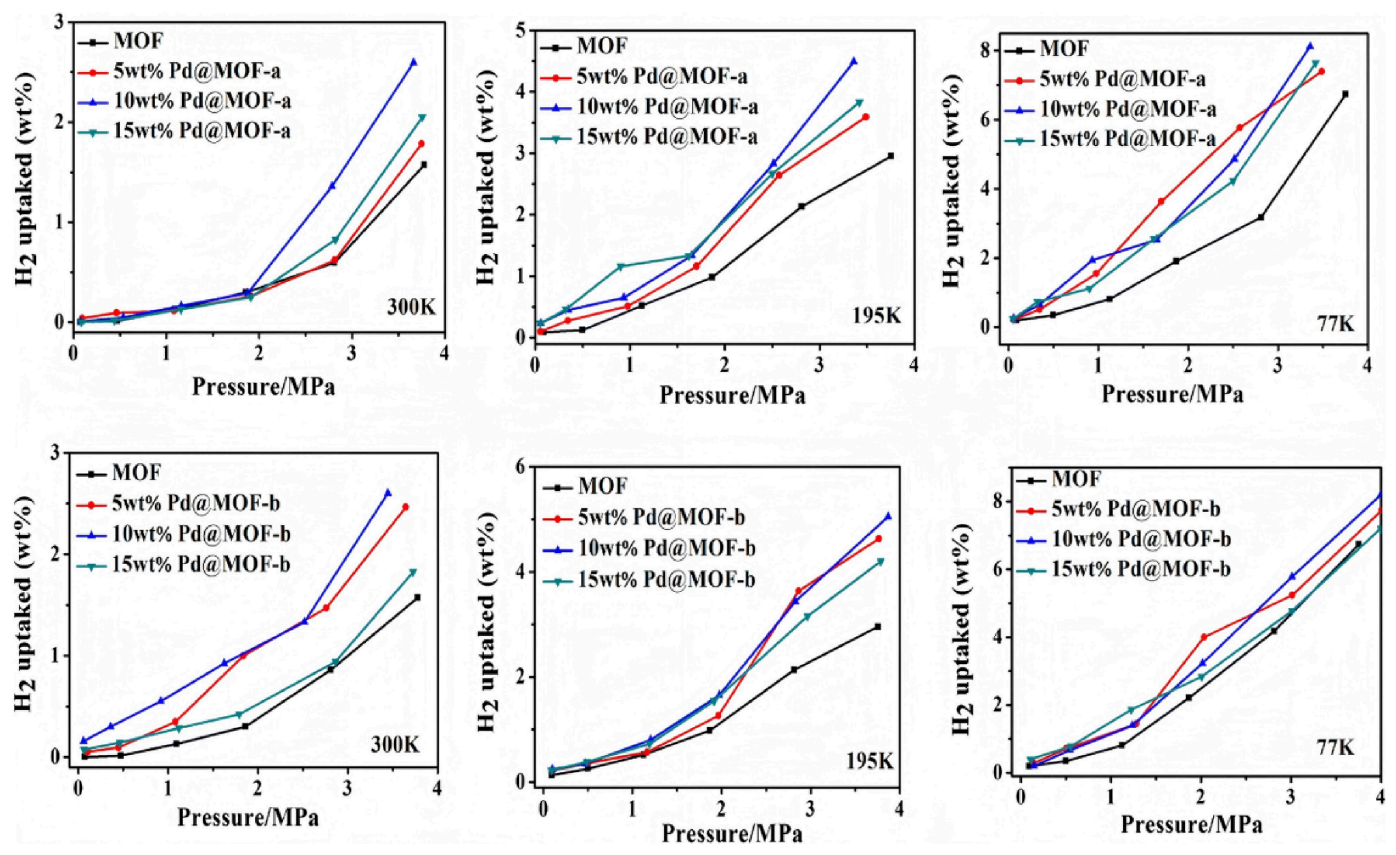
Hydrogen adsorption isotherms at different temperatures (300 K, 195 K and 77 K) for the Pd@MOF-808 series materials [70]. Note: 1 MPa = 10 bar. Reproduced with permission from Xu, J., et al., *J. Mater. Sci.*; published by Springer, 2019.

Various other Zr-based MOFs have also been evaluated for hydrogen storage applications. Naeem et al. [67] synthesized three highly porous Zr(IV)-based MOFs, UBMOF-8, UBMOF-9, and UBMOF-31, using 2,2′-diaminodiphenylmethane dicarboxylic acid, 4,4′-diphenylmethane dicarboxylic acid, and their combinations as ligands. The mixed-linker UBMOF-31 sample exhibited an excellent hydrogen storage capacity of 4.9 wt% at 77 K and 46 bar. Larasati et al. [68] investigated prepared Pd-modified Zr-based MOFs (Pd@ZrBTC) using phenyl tricarboxylic acid as the organic ligand. They found that embedding Pd modified the morphology and crystallinity of the MOF material structure, leading to a smaller specific surface area and pore volume. Unmodified ZrBTC showed the mild-temperature hydrogen storage capacity of 0.018 wt% at 298 K and 1.5 bar. At 373 K and 1.5 bar, the hydrogen storage capacity increased with increasing Pd loading, and 10% Pd@ZrBTC achieved the highest hydrogen storage capacity of 0.56 wt%. Xu et al. [69] synthesized Zr(IV)-based MOFs (MOFs-808) using highly electron-deficient and polar 4,4′-diphenylmethane dicarboxylic acid molecules as the organic ligand. The synthesis conditions (molar ratio of reactants, amount of solvent, reaction temperature, and reaction time) were optimized to obtain a MOFs-808 sample with high crystallinity and specific surface area, which provided a hydrogen storage capacity of 7.31 wt% at 44 K and 70 bar. Xu et al. [70] also prepared Pd-doped MOFs-808 (Pd@MOF-808) samples, which incorporated Pd to influence the specific surface area, enhance the micropore volume, and widen the pore diameter. The influence of the Pd nanoparticle doping amount on the structure and performance of these Pd@MOF-808 materials was then evaluated. At 40 bar and temperatures of 300 K, 195 K, and 77 K, 10 wt% Pd@MOF-808-b exhibited hydrogen storage capacities of 2.61 wt%, 5.04 wt%, and 8.20 wt%, respectively (Figure 6). Table 4 shows some examples of the hydrogen storage performance of various Zr-based MOFs under different temperatures and pressures.

#### 2.1.4. Other Metal-Based MOFs

Many other metal-based MOFs, such as Cr and Fe-based MOFs, have been evaluated for hydrogen storage applications and other fields. For instance, MIL-101(Cr) is a very stable MOF material [26] that exhibits a good hydrogen storage capacity. Yu et al. [71] introduced AC into MIL-101 to prepare an AC-MIL-101(Cr) composite material, which exhibited excellent hydrogen storage performance. At 77 K and 100 bar, the excess hydrogen adsorption capacity of AC-MIL-101(Cr) reached 67.4 mol/kg (13.5 wt%), which was much higher than that of the original MIL-101(Cr) (6.1 wt%) [26]. AC-MIL-101(Cr) exhibits superior hydrogen storage capabilities compared to MIL-100(Cr), which can be attributed to its greater specific surface area [72]. Fe-BTT is synthesized through a high-throughput approach and is characterized by a high density of unsaturated Fe^2+^ sites. The material exhibits a hydrogen storage capacity of 4.1 wt% at 77 K and 95 bar, as well as 1.1 wt% at ambient temperature and 100 bar. The elevated adsorption enthalpy of 11.9 kJ/mol, coupled with the minimal metal–hydrogen interaction distance, indicates that the presence of open metal sites substantially improves the hydrogen storage performance of the MOF material under ambient conditions [73]. Liu et al. [74] introduced a synergistic modification approach involving lithium ion doping combined with graphene oxide (GO) composite integration, which effectively enhanced the mild-temperature hydrogen storage capacity of MIL-100 (Fe) from 0.3 wt% to 2.02 wt% under conditions of 298 K and 50 bar. This improvement represents a significant advancement in the performance of iron-based MOFs for hydrogen storage at ambient temperature during the corresponding timeframe. Kapelewski et al. [75] studied the hydrogen storage performance of various MOFs (including M_2_(m-dobdc) (M=Co, Ni), M_2_(dobdc) (M=Co, Ni), and MOF-5) within the near-ambient temperature range (198–373 K) at different pressures (0–100 bar). The Ni(m-dobdc) had a stronger binding energy for hydrogen gas and showed better hydrogen storage performance than the other hydrogen storage materials. Therefore, at 298 K and 100 bar, this MOF material had a hydrogen adsorption capacity of 2.75 wt%. MOF-76 exhibits remarkable thermal stability; however, its capacity for hydrogen storage is notably limited. At a temperature of 77 K and a pressure of 20 bar, the hydrogen adsorption capacity is observed to be below 1 wt%. This limitation is attributed to the inherent properties of lanthanide-based metal–organic frameworks, which are prone to undergoing structural transformations in their crystal lattice when subjected to thermal or cryogenic conditions [76]. Table 5 shows some examples of the hydrogen storage performance of various MOFs under different temperatures and pressures.

The above survey across Zn, Cu, Zr, and other metal-based MOFs reveals a consistent trend: the highest hydrogen storage capacities are consistently achieved under cryogenic conditions (77 K) across all MOF families. This observation underscores the inherent limitation of physisorption-based hydrogen storage at ambient temperature, where the weak van der Waals interaction between H_2_ molecules and the MOF pore surface cannot achieve the high storage densities required for practical applications. Consequently, the integration of cryogenic temperatures with elevated pressures has emerged as a rational strategy to overcome this limitation. Thermodynamic analysis has confirmed that the 80–120 K temperature range, accessible via mixed-refrigerant Joule–Thomson cooling, offers a practical thermal window for MOF-based hydrogen storage with significantly lower energy consumption than hydrogen liquefaction [42]. Within this regime, the unique structural characteristics of MOFs—high specific surface area, tunable porosity, and abundant pore space—enable hydrogen storage densities approaching those of liquid hydrogen. Gao et al. established a modified Dubinin–Astakhov (D-A) model to simulate hydrogen adsorption isotherms of multiple adsorbents—including MOF-177, MOF-5, Cu_3_(BTC)_2_, MIL-101, and activated carbon AX-21—in a Type III tank configuration across temperatures of 77–298 K and pressures of 1–500 bar [78]. This study quantified the effects of adsorbent bulk density on system-level storage performance and confirmed that cryo-compressed adsorption hydrogen storage can achieve liquid-hydrogen-equivalent densities across multiple temperature ranges [79]. More recently, Xi et al. further extended this line of research by combining experimental measurements with grand canonical Monte Carlo (GCMC) simulations to systematically investigate MIL-101(Cr) under cryo-compressed conditions, specifically targeting its application in Type III composite overwrapped pressure vessels [77]. Their results revealed that MIL-101(Cr) achieves a hydrogen uptake of 9.86 wt% at 77 K and 180 bar, and 12.27 wt% at 77 K and 700 bar. Crucially, when packed into a Type III tank at the engineering scale, the MIL-101(Cr)-filled system delivers a usable hydrogen storage capacity of 8.07 wt% under 77 K and 700 bar conditions [77]. These findings collectively establish that the combination of MOFs with cryo-compressed storage conditions represents a practically viable pathway toward high-density hydrogen storage. The applicability of this cryo-compressed strategy has also been demonstrated in other porous frameworks. Gao et al. recently reported a simulation study on COFs under cryo-compressed conditions across a wide pressure range [80].

### 2.2. Factors Affecting Hydrogen Storage Performance of MOFs

#### 2.2.1. Specific Surface Area, Pore Volume, and Pore Size

**Figure 7:**
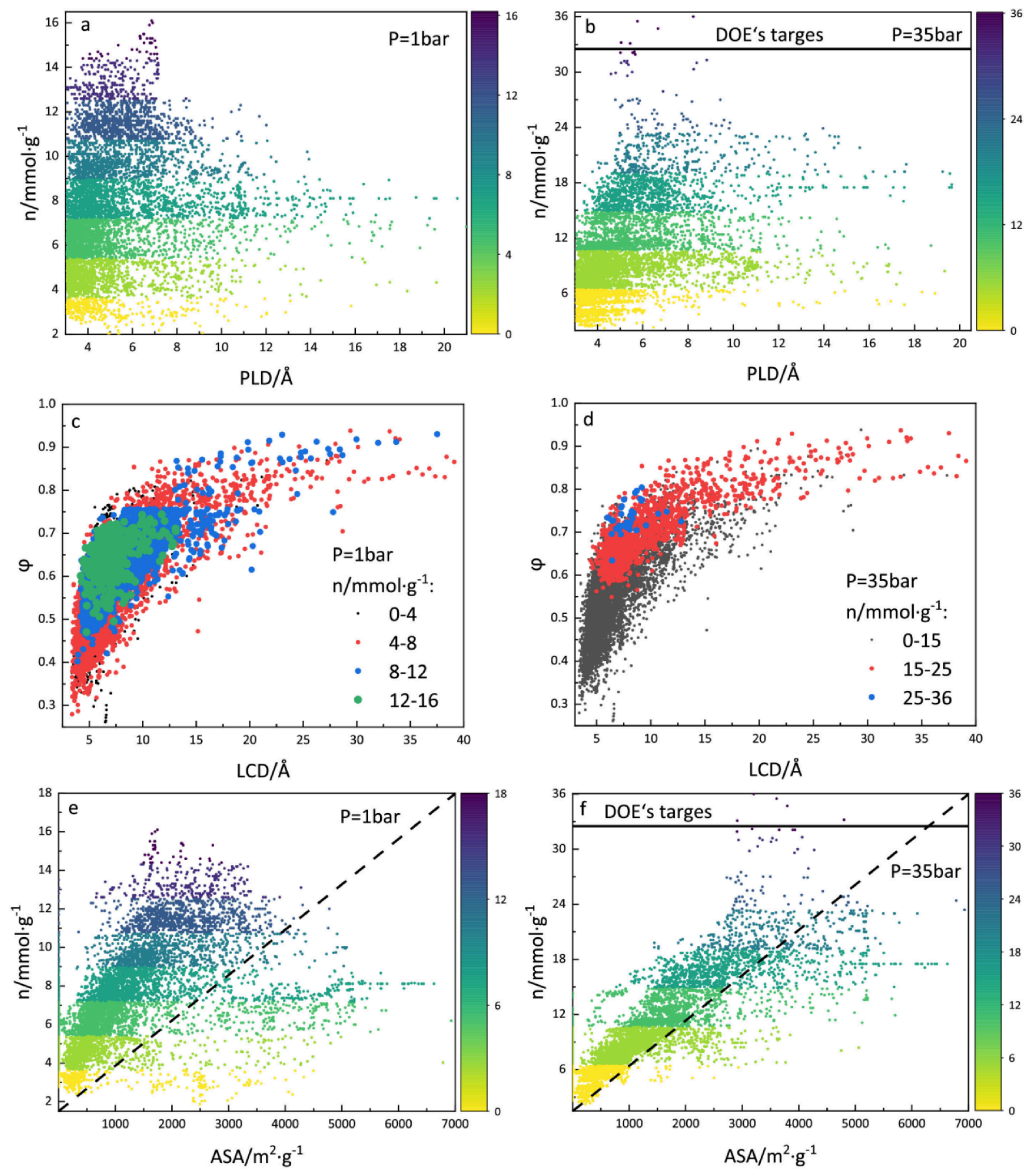
Relationship among pore size, porosity (**a**–**d**), surface area (**e**,**f**), and hydrogen adsorption capacities of MOFs at 1 and 35 bar [82]. Reproduced with permission from Zhang, X., Zheng, Q. R., He, H. Z., *J. Taiwan Inst. Chem. Eng.*; published by Elsevier, 2022.

At low pressures, H_2_ adsorption is affected by the pore diameter, but at low temperature and high pressures, the H_2_ adsorption capacity depends on the specific surface area [79]. Yang et al. established the relationship between the hydrogen storage capacity of mechanochemically synthesized Cu_3_(BTC)_2_ and BET specific surface area, Langmuir specific surface area, and effective pore volume [81]. Figure 7 illustrates the relationship among pore size, porosity, surface area, and hydrogen adsorption capacities of MOFs at 1 and 35 bar [82]. A larger specific surface area and pore volume can provide more hydrogen adsorption sites, leading to a larger hydrogen storage capacity [53]. It is well known that adsorbent surfaces typically have relatively low interaction energies with hydrogen [83]. However, if the pores of the adsorbent are small enough such that the potential fields from opposite walls overlap, the interaction potential can be enhanced. Therefore, adsorbents with pore diameters close to the molecular diameter of H_2_ can exhibit enhanced interactions with hydrogen [84]. In addition, having the right pore size helps hydrogen molecules enter and stick to the surface more effectively [2,85,86]. When the pore diameter matches the dynamic diameter of hydrogen molecules, the pore channels can be more effectively filled with hydrogen, improving the hydrogen storage density. Excessively large pore diameters lead to weak interactions between hydrogen molecules and the pore walls, but if the pore diameters are too small, hydrogen molecules cannot easily enter the pore channels.

#### 2.2.2. Metal Ion Doping

Different metal ions have different electronic structures and chemical properties, which will affect the interaction strength between MOFs and hydrogen molecules [87]. Theoretical analyses have further substantiated that lithium doping markedly improves the hydrogen storage capabilities of aluminum-based MOF-519. At a temperature of 77 K and a pressure of 1 bar, the hydrogen storage capacity of Li-doped MOF-519 reaches 2.132 wt%, representing an enhancement of 6.24% relative to the undoped MOF-519 [88]. Chen et al. [89] prepared MFU-4l-Li via post-synthetic metal exchange (Li^+^ doping), which exhibited a hydrogen storage capacity of 9.9 wt% at 77 K and 100 bar, demonstrating excellent hydrogen storage performance. Yao et al. [90] employed a divalent metal doping strategy, incorporating ions such as Mg^2+^ and Cu^2+^, to functionalize the highly stable MOF-808 framework. Notably, the Mg-doped MOF-808@Mg demonstrated a hydrogen adsorption capacity of 1.3 wt% at 77 K and 1 bar, corresponding to a 33% enhancement relative to the pristine MOF-808. Similarly, the Cu-doped MOF-808@Cu exhibited a hydrogen uptake of 0.916 wt% under identical conditions, reflecting an approximate 12% improvement over the undoped material. This metal doping approach preserves the inherent stability of Zr-based MOFs while introducing effective open metal sites, thereby offering a viable strategy for the development of MOFs that synergistically combine robust stability with enhanced hydrogen storage capabilities. The above research results indicate that doping with metal ions can effectively enhance the hydrogen storage capacity of MOFs.

#### 2.2.3. Ligands

The architecture of MOFs is fundamentally influenced by the geometry and connectivity of their constituent ligands [91]. A diverse array of organic ligands is commonly employed in MOF synthesis, encompassing dicarboxylates such as malonate, oxalate, succinate, glutarate, and terephthalate; tricarboxylates including trimesate and citrate; as well as azole-based ligands like pyrazole and 1,2,3-triazole. The functionalization of these organic ligands represents a strategic approach to improve hydrogen storage capabilities within MOFs, achieved through the incorporation of various functional groups such as amino (-NH_2_), hydroxyl (-OH), methyl (-CH_3_), and pyridyl moieties. For instance, Han et al. [92] functionalized UiO-66 by introducing dihydroxy, dimethoxy, and diethoxy groups, producing modified materials UiO-66-(OH)_2_, UiO-66-(OCH_3_)_2_, and UiO-66-(OCH_2_CH_3_)_2_. The introduction of these functional groups altered the electronic density distribution of the ligands, optimized the pore structure of the MOFs, and enhanced the materials’ affinity for hydrogen adsorption. The diethoxy-modified UiO-66-(OCH_2_CH_3_)_2_ achieved the best performance among the four materials, reaching 4.56 wt% hydrogen uptake at 77 K and 40 bar. Similarly, Prasetyo et al. [93] confirmed through DFT that fluorine substitution of hydroxyl groups can significantly enhance the adsorption of H_2_ by MOF-801. After fluorination, the theoretical gravimetric hydrogen storage capacity of MOF-801 can reach 1.1–1.4 wt%, which is higher than that of the unmodified sample. The enhancement mechanism originates from the polarization effect caused by the high electronegativity of the F atoms, making the Zr–F sites efficient centers for H_2_ adsorption. Overall, rational ligand functionalization is therefore an effective strategy to develop high-performance hydrogen storage MOFs.

### 2.3. Challenges for MOFs in Hydrogen Storage

#### 2.3.1. Synthetic Costs and Scalable Production

MOFs are novel porous materials with promising applications, but their high cost and identifying the most cost-effective manufacturing routes for industrial commercialization still poses major technical and economic challenges that require further study [94]. The cost structure of MOF production is dominated by several factors: expensive organic ligands, metal precursors, organic solvents, and energy-intensive synthesis conditions [95]. Recent techno-economic assessments have shown that even at pilot scale, MOF production costs remain substantially higher than those of conventional porous materials such as zeolites, underscoring the need for cost reduction strategies [95,96].

When noble metals such as Pt are employed as dopants to enhance hydrogen storage performance via spillover effects, their high unit prices raise legitimate economic concerns. However, the actual loading levels in MOF composites are typically very low, with metal nanoparticles dispersed exclusively on the external surface or within pore channels, resulting in minimal noble metal content per unit mass of composite [40,45]. For instance, Lee et al. used only 5 wt% Pt on activated carbon in MOF-5 composites, achieving a hydrogen storage capacity of 2.3 wt% at 298 K and 100 bar [45]. Kang et al. further demonstrated that 0.71 wt% hydrogen uptake at 300 K could be achieved through spillover using Pt-decorated UiO-66, with the Pt loading representing a negligible fraction of the total material weight [40]. These examples indicate that the impact of noble metal costs on the overall material price can be limited through controlled and minimal loading.

An additional challenge in MOF synthesis is that many reaction conditions must be precisely controlled, including the temperature, pressure, type and dosage of solvents, reaction time, and pH value. Even a slight change in these conditions can have a significant impact on the structure, purity, and performance of the final product, meaning that MOFs synthesis procedures often exhibit poor reproducibility and stability. For example, in the synthesis of some MOFs, fluctuations in temperature may lead to uneven crystal growth rates, resulting in the formation of defects or impurity phases. This significantly affects the final product quality.

Currently, most existing MOFs synthesis methods are suitable for small-scale laboratory production and cannot be easily scaled up into large-scale industrial production processes [97]. Attempts at scaling up these processes lead to various difficulties [96] (e.g., ensuring the uniformity of the reaction system, mitigating reductions in heat and material transfer efficiency), which can result in lower product quality, lower yields, and a sharp increase in costs. For example, large-scale reactors typically have lower mixing efficiencies compared to small-scale laboratory reactions, resulting in local concentration inhomogeneities that affect the MOFs crystal growth process.

The development of mild, precisely controllable, and scalable synthesis routes is essential for broader industrial adoption. Approaches such as mechanochemical synthesis, flow chemistry, microwave-assisted methods, and water-based green synthesis have demonstrated significant potential for reducing both capital and operational costs [98]. Nevertheless, critical challenges remain in ensuring product uniformity, heat and mass transfer efficiency, and reproducibility upon scale-up. Future efforts should prioritize the integration of low-cost precursor strategies, green synthesis methodologies, and comprehensive techno-economic analysis to enable the commercial translation of MOF-based hydrogen storage materials.

#### 2.3.2. Stability

The structural stability of MOFs is a critical parameter determining their practical applicability [99]. Currently, no neat MOFs have simultaneously satisfied the full set of requirements—stability, reusability, low cost, and high efficiency—for ambient hydrogen storage [100].

Mechanical stability is a major concern. MOFs are difficult to utilize in powder form, and after forming, MOFs may undergo pulverization under extreme service conditions (e.g., cryogenic temperature, high pressure, or rapid hydrogen charge–discharge cycling). Under low-temperature and high-pressure conditions, the crystal structure of MOFs may be subjected to large stress, which can distort or break the coordination bonds between the metal ions/clusters and organic ligands of the MOF structure. This can destroy the integrity of the MOF crystal structure, inducing gradual pulverization. During rapid hydrogen gas charging and discharging, the adsorption and desorption of hydrogen will cause volume changes in MOFs. Repeated volume expansion and contraction generate mechanical stress, which causes pulverization once exceeding the structural tolerance of MOFs. Therefore, MOFs with improved mechanical strength and stability that exhibit strong interactions with hydrogen gas and good anti-pulverization performance must be developed for their utilization in hydrogen storage applications. It should be noted, however, that the above considerations are based primarily on isothermal pressure-cycling conditions. In practical MOF-based hydrogen storage systems, the operating cycle involves not only pressure swings but also repeated temperature swings between cryogenic adsorption (77 K) and near-ambient desorption conditions, which introduce additional thermo-mechanical stresses.

The knowledge gap regarding multi-cycle temperature-swing stability is increasingly recognized as a critical barrier to the deployment of MOFs in hydrogen storage. Peng et al. highlighted that each cryogenic-to-ambient thermal cycle can induce interfacial stress between the metal nodes and organic linkers due to differential thermal expansion, and repeated cycling may lead to micro-crack accumulation and eventual framework fatigue [101]. Similarly, the U.S. Department of Energy’s updated Hydrogen Program Plan (2024) [13] identified the study of material compatibility with hydrogen under combined cryogenic and high-pressure conditions as a core R&D priority for hydrogen storage, underscoring the need to develop hydrogen-compatible materials with assured durability and safety under realistic cyclic service conditions [13]. Despite this growing recognition, systematic experimental investigations into the long-term cycling stability of MOFs under coupled temperature-pressure swing conditions (77 K→298 K, 1→700 bar) remain scarce. Future research efforts should prioritize the establishment of standardized cyclic stability testing protocols that simulate the actual thermal and pressure trajectories of MOF-based hydrogen storage systems, thereby bridging the critical gap between laboratory material characterization and practical engineering requirements.

#### 2.3.3. Computational Simulation Studies on MOFs for Hydrogen Storage

Research on hydrogen storage within MOFs, via computational simulations and machine learning techniques, has progressed from isolated adsorption modeling to a comprehensive methodological framework [102,103]. This integrated framework combining high-throughput screening, mechanistic analysis and rational material design has become a core strategy to overcome performance bottlenecks of hydrogen storage materials and cut experimental costs.

Grand Canonical Monte Carlo (GCMC) simulation is widely recognized as the standard quantitative approach for evaluating hydrogen storage capacity, adsorption sites, and thermodynamic properties of MOFs [104]. This method enables precise prediction of gravimetric and volumetric hydrogen storage capacities, usable storage amounts, and adsorption isotherms across varying temperature and pressure conditions. In a study by Granja-DelRío et al. [105], the GCMC technique was employed to investigate the Zn(II)/Cd(II)-based MRT series of MOFs, revealing that MRT2 and MRT4 surpass the hydrogen storage performance targets set by the U.S. Department of Energy (DOE) at 77 K and approximately 50 bar. Furthermore, their findings indicated notable competitiveness at ambient temperature, achieving onboard driving ranges comparable to those of high-pressure hydrogen storage systems but operating at reduced pressures. Additionally, Zhang et al. [86] integrated GCMC simulations with high-throughput screening of 7622 MOFs to identify structural parameters conducive to efficient room-temperature hydrogen storage, thereby offering direct structural guidelines to inform experimental synthesis efforts.

Density Functional Theory (DFT) focuses on adsorption energy, interaction mechanisms, and electronic interactions, revealing the microscopic binding rules between MOFs and hydrogen. Zhao et al. [106] compared the hydrogen adsorption energies of MOF-808 and Ce-MOF-808 through DFT calculations, confirming that Ce doping enhances the adsorption strength, providing an energetic basis for composite material design. Yu et al. [107] conducted a combined DFT and GCMC study on MOF-650, achieving a quantitative analysis correlating pore structure, metal sites, and hydrogen storage performance.

**Figure 8:**
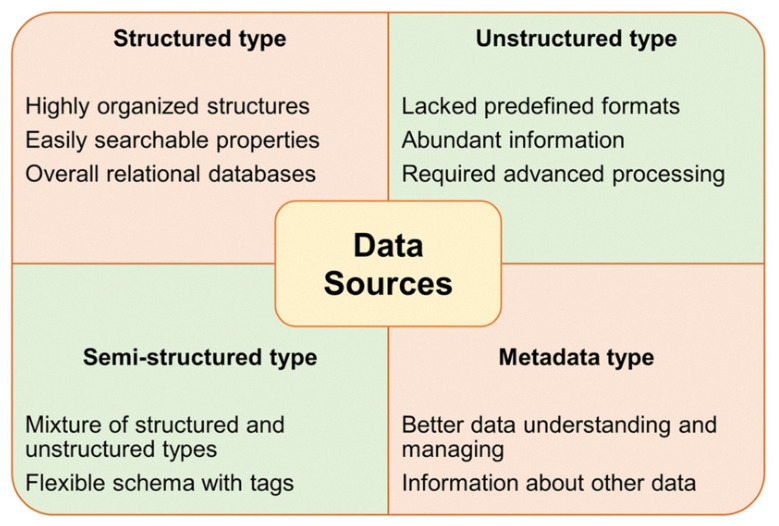
Four types of data for machine learning [108]. Reproduced with permission from Han, et al., *Chem. Soc. Rev.*; published by Royal Society of Chemistry, 2025.

With the continuous accumulation of MOF structures and the improvement of computational methods, the scale of computational research has been further expanded from single-material simulation to large-scale database-oriented high-throughput screening and data-driven material discovery. The four types of data that a database can be composed of are structured, unstructured, semi-structured, and metadata (Figure 8) [108]. In response to the exponential expansion of the MOFs structural database, high-throughput computational screening has emerged as a pivotal approach for the rapid identification of high-performance hydrogen storage materials [109]. Ahmed et al. [110] performed GCMC high-throughput screening on nearly 500,000 MOFs, identifying materials such as PCN-610/NU-100 that exhibited a usable hydrogen storage capacity of 13.9 wt% under temperature and pressure swing conditions of 77 K at 100 bar and 160 K at 5 bar. Similarly, Chen et al. [111] conducted extensive simulations on 98,695 MOFs derived from the HyMARC database, thereby generating high-quality labeled datasets to support the development of machine learning models.

Conventional machine learning approaches have demonstrated high predictive accuracy; however, they often lack adherence to physical principles, frequently yielding outputs that contravene established adsorption laws. To address this limitation, Chen et al. [112] developed a physics-informed neural network (PINN) model that integrates monotonic relationships among crystal density, specific surface area, pore volume, and hydrogen storage capacity directly into the loss function. This approach facilitated the rapid screening of 820,000 MOFs under variable pressure conditions at 77 K and pressures ranging from 5 to 100 bar. Building upon this, Zhang et al. [82] applied a gradient boosting regression (GBR) algorithm to extrapolate low-temperature, high-pressure hydrogen storage capacities from room-temperature, low-pressure data, thereby expanding the database from 418 to 8024 MOFs types. The integration of machine learning with experimental validation has been further highlighted in a recent review by Dubey et al. [113], which emphasized the potential of synergistic experimental and computational approaches for accelerating MOFs discovery.

In summary, computational simulations and machine learning have significantly advanced the rational design and high-throughput screening of MOFs for hydrogen storage, enabling efficient prediction of adsorption behavior, structure–performance relationships, and material discovery at reduced experimental cost [114]. Nevertheless, critical challenges remain to be addressed for more reliable and practical predictions. Future research should emphasize the integration of structural defects, thermal effects, and realistic synthesis conditions into computational models. Meanwhile, the combination of physics-constrained machine learning, multiscale simulation, and closed-loop validation between theory and experiment is highly desirable to bridge the gap between theoretically promising structures and practically applicable hydrogen storage MOFs.

## 3. Prospects and Suggestions

MOFs for hydrogen storage is evolving from fundamental investigation toward practical implementation. It is important to highlight that MOFs demonstrate superior hydrogen storage capabilities under conditions of low temperature (77 K) and high pressure, attributable to strengthened van der Waals interactions and the dense packing of hydrogen molecules within their porous structures. In contrast, hydrogen storage technologies at relatively mild temperatures are more in line with practical application needs and are also the current research focus in this field. This approach faces significant challenges, including inherently weak interactions between hydrogen molecules and the framework, as well as relatively low adsorption enthalpy, which collectively constrain its effectiveness.

Future advancements are anticipated to concentrate on three primary objectives: achieving efficient hydrogen storage under relatively mild temperature, enabling cost-effective large-scale synthesis, and employing intelligent design strategies integrated with multi-disciplinary coupling. Meanwhile, enhancing the structural stability of MOFs is also crucial. Strategies such as incorporating rigid building units to reinforce framework stability and applying protective surface coatings via post-modification can effectively improve the mechanical strength and anti-pulverization performance of MOFs under practical working conditions. Nevertheless, as highlighted in Section 2.3.2, the long-term stability of MOFs under coupled temperature-pressure swing conditions—the actual operating mode of cryo-adsorptive hydrogen storage—remains largely unexplored. Systematic experimental investigations into multi-cycle thermal stability between cryogenic and ambient temperatures are urgently needed to establish the performance baselines required for practical deployment.

From an application standpoint, MOFs exhibit distinct and promising potential across three specific industrial contexts. First, MOFs demonstrate considerable promise for use in cryo-compressed hydrogen refueling stations. Economic analysis has shown that cryo-compressed hydrogen storage and transportation is more advantageous than compressed gaseous hydrogen at normal electricity prices and for short-to-medium transportation distances, providing a favorable economic context for MOF-coupled storage systems [12,17]. By integrating MOFs into the storage tanks, the required operating pressure can be further reduced, potentially lowering the system capital cost and enhancing the competitiveness of cryo-compressed hydrogen infrastructure. Second, MOFs are particularly well-suited for high-value logistics and specialized transportation sectors, including hydrogen-powered drones, forklifts, and medical transport vehicles. Additionally, cryo-compressed hydrogen storage has been recognized as a promising pathway for long-haul heavy-duty truck transportation in national hydrogen development roadmaps [13,15]. In these cases, volumetric efficiency and safety considerations often take precedence over gravimetric weight. The capacity of MOFs to densify hydrogen at moderate pressures facilitates a compact design and improved safety profile, thereby justifying the associated material costs within these niche markets. Lastly, stationary hydrogen storage for grid balancing constitutes another practical application. Unlike mobile systems, stationary storage solutions are less constrained by the weight of the storage medium. By coupling MOFs with industrial waste heat recovery or cost-effective cooling technologies, operational expenditures can be reduced, rendering MOF-based storage a competitive alternative for long-duration energy storage applications. Future research should focus not only on lowering synthesis costs but also on tailoring MOFs properties—such as thermal conductivity and mechanical stability—specifically for these targeted applications to maximize their techno-economic potential.

## Figures and Tables

**Table 1:** Hydrogen storage performance of representative porous adsorbents.

MOFs	Category	Temperature (K)	Pressure (bar)	Gravimetric H_2_ Uptake (wt%)	Reference
MOF-5	MOF	78	1	4.50	[23]
MOF-177	MOF	77	70	7.50	[24]
MOF-210	MOF	77	60	17.70	[25]
MIL-101(Cr)	MOF	77	80	6.10	[26]
COF-102	COF	77	35	7.20	[27]
COF-103	COF	77	35	7.20	[27]
Li@TPB-DMTP-COF	COF	77	80	6.91	[28]
3D-F-COF	COF	77	90	5.96	[20]
MSC-30	Activated carbon	77	40	5.80	[29]
AX-21	Activated carbon	77	40	6.00	[30]
LTA zeolite	Zeolite	77	-	4.80	[31]

**Table 2:** Hydrogen storage performance of various Zn-based MOFs.

MOFs	Metal Ion	Ligand	Temperature (K)	Pressure (bar)	Gravimetric H_2_ Uptake (wt%)	Reference
MOF-5	Zn^2+^	1,4-benzenedicarboxylate	78	20	4.50	[23]
Pt-ACs-MOF-5	Zn^2+^	1,4-benzenedicarboxylate	298	100	2.30	[45]
P-MOF	Zn^2+^	1,4-benzenedicarboxylate	77	1	1.20	[46]
N-MOF	Zn^2+^	1,4-benzenedicarboxylate	77	1	2.00	[46]
Ni-MOF-5	Zn^2+^	1,4-benzenedicarboxylate	77	1	1.53	[47]
Co-MOF-5	Zn^2+^	1,4-benzenedicarboxylate	77	1	1.53	[47]
Fe-MOF-5	Zn^2+^	1,4-benzenedicarboxylate	77	1	0.99	[47]
MOF-177	Zn^2+^	1,3,5-benzenetribenzoate	77	70	7.50	[48]
			298	100	0.62	
MOF-210	Zn^2+^	4,4′,4″-[benzene-1,3,5-triyl-tris(benzene-4,1-diyl)]tribenzoate	77	60	17.60	[25]
Li-ZIF-70	Zn^2+^	imidazolate (Im^−^); 2-nitroimidazolate (nIm^−^)	298	100	3.08	[49]
Na-ZIF-70	Zn^2+^	imidazolate (Im^−^); 2-nitroimidazolate (nIm^−^)	298	100	2.19	[49]
ZIF-8@ZIF-67	Zn^2+^	2-methylimidazolate (mIm^−^); 2-methylimidazolate (mIm^−^)	77	1	2.03	[51]
ZIF-67@ZIF-8	Zn^2+^	2-methylimidazolate (mIm^−^); 2-methylimidazolate (mIm^−^)	77	1	1.69	[51]

**Table 3:** Hydrogen storage performance of various Cu-based MOFs.

MOFs	Metal Ion	Ligand	Temperature (K)	Pressure (bar)	Gravimetric H_2_ Uptake (wt%)	Reference
HKUST-1	Cu^2+^	1,3,5-benzenetricarboxylate	77	80	3.60	[52]
Cu-BTC/Ni@f-MWCNTs	Cu^2+^	1,3,5-benzenetricarboxylate	298	70	4.68	[54]
Cu-BTC/Pd@f-MWCNTs	Cu^2+^	1,3,5-benzenetricarboxylate	298	70	5.31	[54]
Cu-BTC/GO	Cu^2+^	1,3,5-benzenetricarboxylate	298	1	0.46	[55]
			298	3	0.49	
PCN-6′	Cu^2+^	1,3,5-tris(3,5-dicarboxyphenyl)benzene	77	30	7.00	[56]
			298	100	1.30	
Cu_3_(BTC)_2_	Cu^2+^	1,3,5-benzenetricarboxylate	77	1	2.18	[57]
NOTT-103	Cu^2+^	3,3′,5,5′-tetracarboxybiphenyl	77	60	7.78	[58]
NU-2100	Cu^+^	1,5-dihydrobenzo [1,2-d:4,5-d’]bis([1,2,3]triazole)	233–296	5–100	4.8	[59]

**Table 4:** Hydrogen storage performance of various Zr-based MOFs.

MOFs	Metal Ion	Ligand	Temperature (K)	Pressure (bar)	Gravimetric H_2_ Uptake (wt%)	Reference
UiO-66	Zr^4+^	1,4-benzenedicarboxylate	77	18	3.35	[62]
UiO-66(H_2_ADC)-SS	Zr^4+^	Alkyldicarboxylate	298	50	1.09	[64]
UiO-66(H_2_ADC)-S	Zr^4+^	Alkyldicarboxylate	298	50	2.99	[64]
Pt/aUiO-Ac	Zr^4+^	1,4-benzenedicarboxylate	303	30	0.38	[40]
Pt/aUiO-Cl	Zr^4+^	1,4-benzenedicarboxylate	303	30	0.71	[40]
UiO-66-AO@Si	Zr^4+^	1,4-benzenedicarboxylate	77	2.1	0.35	[65]
UiO-66-SO_3_H@Si	Zr^4+^	1,4-benzenedicarboxylate	77	2.1	0.12	[65]
UiO-66-PDCA	Zr^4+^	1,4-benzenedicarboxylate	77	2.1	0.25	[65]
Cu@UiO-66	Zr^4+^	1,4-benzenedicarboxylate	298	60	0.26	[66]
Ni@UiO-66	Zr^4+^	1,4-benzenedicarboxylate	298	60	0.45	[66]
CuNi@UiO-66	Zr^4+^	1,4-benzenedicarboxylate	298	60	0.74	[66]
UBMOF-31	Zr^4+^	1,4-benzenedicarboxylate	77	46	4.90	[67]
MOFs-808	Zr^4+^	1,3,5-benzenetricarboxylate	44	70	7.31	[69]
Pd@MOF-808	Zr^4+^	1,3,5-benzenetricarboxylate	300	40	2.61	[70]
			195	40	5.04	
			77	40	8.20	

**Table 5:** Hydrogen storage performance of various MOFs.

MOFs	Metal Ion	Ligand	Temperature (K)	Pressure (bar)	Gravimetric H_2_ Uptake (wt%)	Reference
AC-MIL-101(Cr)	Cr^3+^	1,4-benzenedicarboxylate	77	100	13.50	[71]
Fe-BTT	Fe^2+^/Fe^3+^	1,3,5-benzenetristetrazolate	77	95	4.10	[73]
MIL-100(Fe)/GO	Fe^3+^	1,3,5-benzenetricarboxylate	298	50	2.02	[74]
Ni(m-dobdc)	Ni^2+^	4,6-dioxido-1,3-benzenedicarboxylate	298	100	2.75	[75]
MOF-76	Nd^3+^	1,4-benzenedicarboxylate	77	20	1.00	[76]
MIL-101(Cr)-filled Type III tank	Cr^3+^	1,4-benzenedicarboxylate	77	700	12.27	[77]

## Data Availability

No new data were created or analyzed in this study.
